# Rapid, simple and direct detection of *Meloidogyne hapla* from infected root galls using loop-mediated isothermal amplification combined with FTA technology

**DOI:** 10.1038/srep44853

**Published:** 2017-04-03

**Authors:** Huan Peng, Haibo Long, Wenkun Huang, Jing Liu, Jiangkuan Cui, Lingan Kong, Xianqi Hu, Jianfeng Gu, Deliang Peng

**Affiliations:** 1 State Key Laboratory for Biology of Plant Diseases and Insect Pests, Institute of Plant Protection, Chinese Academy of Agricultural Sciences, Beijing 100193, China; 2 Environment and Plant Protection Institute, Chinese Academy of Tropical Agricultural Sciences, Haikou, Hainan 571101, China; 3 The National Engineering Research Center of Agribiodiversity Applied Technologies, Yunnan Agricultural University, Kunming 650201, China; 4 Academy of Inspection and Quarantine, Ningbo, Zhejiang, 315012, China

## Abstract

The northern root-knot nematode (*Meloidogyne hapla*) is a damaging nematode that has caused serious economic losses worldwide. In the present study, a sensitive, simple and rapid method was developed for detection of *M. hapla* in infested plant roots by combining a Flinders Technology Associates (FTA) card with loop-mediated isothermal amplification (LAMP). The specific primers of LAMP were designed based on the distinction of internal transcribed spacer (ITS) sequences between *M. hapla* and other *Meloidogyne* spp. The LAMP assay can detect nematode genomic DNA at concentrations low to 1/200 000, which is 100 times more sensitive than conventional PCR. The LAMP was able to highly specifically distinguish *M. hapla* from other closely related nematode species. Furthermore, the advantages of the FTA-LAMP assay to detect *M. hapla* were demonstrated by assaying infected root galls that were artificially inoculated. In addition, *M. hapla* was successfully detected from six of forty-two field samples using FTA-LAMP technology. This study was the first to provide a simple diagnostic assay for *M. hapla* using the LAMP assay combined with FTA technology. In conclusion, the new FTA-LAMP assay has the potential for diagnosing infestation in the field and managing the pathogen *M. hapla*.

Root-knot nematodes (*Meloidogyne* spp.) are one of the most economically damaging genera of plant-parasitic nematodes in horticultural and field crops. They are distributed worldwide, infect more than 2000 plant species and reduce the global crop yields by approximately 5%^1^. The genus has more than 90 species, including the four *Meloidogyne* species of *M. hapla, M. incognita, M. javanica* and *M. arenaria*, which are major pests worldwide^2^. Northern root-knot nematode (*M. hapla*) has a broad range of hosts and reproduces on tomato, potato, carrots, alfalfa, onion and many other plants, which causes substantial reduction of crop yield and quality^3^,^4^. *M. hapla* can presumably withstand colder temperatures and can occasionally be found in the cooler upland tropics^5^, in contrast, the other three species were adapted to areas with high temperatures. In China, this species has been found in more than 10 provinces^6^, and the distribution range is increasingly widespread.

Traditionally, identification of *Meloidogyne* species had been performed based on morphological characters of second-stage juveniles, perineal patterns of adult females^7^ and isozyme phenotypes^8^. Isozymes are highly reliable for identifying the root-knot nematode, but measurement of isozymes requires examining of adult females as well as considerable skills, furthermore, it is time-consuming^9^. Many different DNA-based methods have been reported for the identification of a large number of *Meloidogyne* spp^10^. Random amplified polymorphism DNA (RAPD) was used to distinguish *M. hapla* from other root-knot nematodes^11^. Species-specific sequence-characterized amplified region (SCAR)^12^, satellite DNA^13^, ribosomal DNA^14^,^15^,^16^,^17^ and high-resolution melting curve (HRMC)^18^ were also employed for the detection of *M. hapla*.

The loop-mediated isothermal amplification (LAMP) assay, originally developed by Notomi *et al*.^19^, is a simple and rapid method that allows DNA amplification under isothermal conditions. The technique can amplify DNA with high specificity and sensitivity under isothermal conditions within 1 hour based on the auto-cycling strand displacement DNA synthesis by *Bst* DNA polymerase^19^. LAMP utilizes four specific primers that were designed by six different regions of the target gene. The LAMP assay does not require much technical skill but a thiermal cycler. Additionally, the LAMP product can be seen with the naked eye by adding SYBR Green I, according to the colour change and the lateral flow dipstick (LFD). Because it is high efficient and does not require special laboratory facilities, this assay has recently shown promising in the specific and rapid detection of clinical^20^,^21^ and agricultural pathogens^22^,^23^,^24^. However, in plant parasitic nematodes, only *Bursaphelenchus xylophilus*^25^, *M. enterolobii*^9^, *M. incognita*^26^ *Radopholus similis*^27^ and *Tylenchulus semipenetrans*^28^ have been detected by LAMP. In this study, we established a sensitive and specific LAMP method for the direct detection of *M. hapla* from infected plant root galls based on the rDNA-ITS.

## Results

### Primer design and reaction optimization

**Figure 1:**
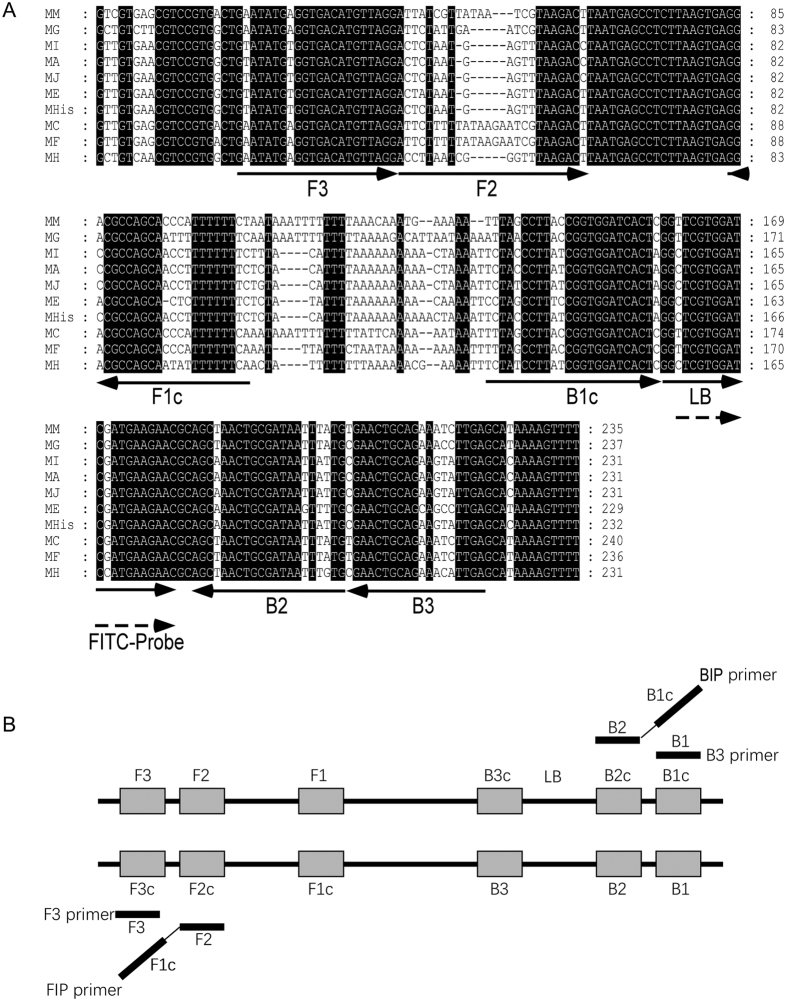
Primers designed for *M. hapla* LAMP assay based on rDNA-ITS sequences. Arrows represent the location of the primers. FIP consisted of the F1c and F2 sequence, BIP consisted of B1c and B2 sequence. MM, *Meloidogyne minor*, MG, *Meloidogyne graminicola*, MI, *Meloidogyne incognita*, MA, *Meloidogyne arenaria*, MJ, *Meloidogyne javanica*, ME, *Meloidogyne enterolobii*, MHis, *Meloidogyne hispanica*, MC, *Meloidogyne chitwoodi*, MF, *Meloidogyne fallax* and MH, *Meloidogyne hapla*.

The specific primers were designed for LAMP based on the sequence dissimilarity among *M. hapla* and other closely related *Meloidogyne* species at the primer positions, and the primer sets with high efficiency and low false positive rate were selected (Fig. 1 and Supplemental Table 1). The LAMP reaction was optimized under the conditions of 5.0 mmol∙L^−1^ Mg^2+^ and 2.4 mmol∙L^−1^ dNTPs, without betaine at 65 °C for 45 min.

### Detection and confirmation of LAMP products

**Figure 2:**
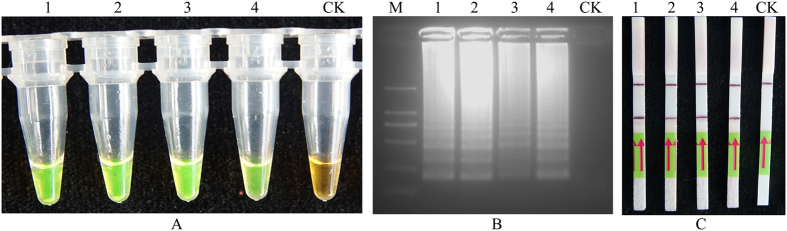
Loop-mediated isothermal amplification of DNA (LAMP) for detection of *M. hapla*. (**A**), LAMP products were visually observed with SYBR Green I fluorescence dye. (**B**), 2% agarose Gel electrophoresis separation of LAMP products. (**C**), A lateral flow strips (LFD) detection system was used to detect LAMP amplification. Lines 1–4 represent the *M. hapla* isolates Mh1, Mh2, Mh3, Mh4, CK, which was the negative control with no template DNA. M represents a DL2000 DNA size marker.

The LAMP products were detected by adding SYBR Green I fluorescence dye. After amplifications followed by adding SYBR Green I fluorescence dye, the tubes containing *M. hapla* samples produced positive reactions that the solution appeared green, while the solution remained orange in the negative reactions (Fig. 2A). The LAMP products were separated using two percent agarose gel electrophoresis, and the bands presented ladder-shaped characteristic (Fig. 2B). To eliminate the false positive interference, the LAMP reaction products were evaluated by lateral flow strips (LFD): the sample with positive amplification showed both control and test lines, whereas the negative control only displayed the control line (Fig. 2C).

### Specificity of LAMP assay

**Figure 3:**
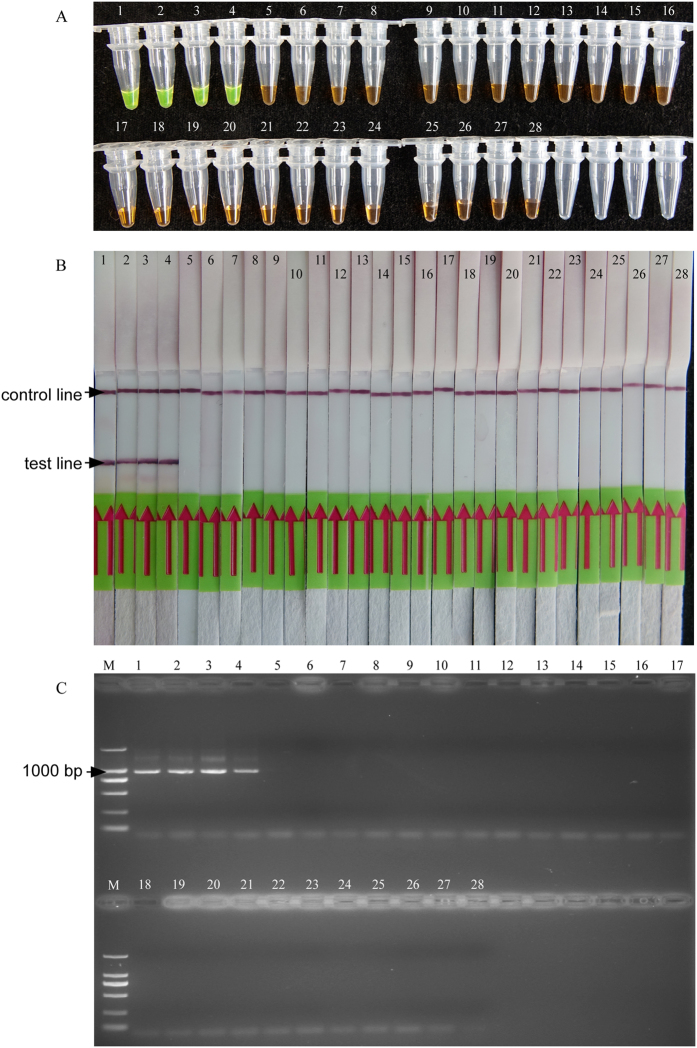
Specificity results of LAMP and traditional PCR with reactions contain *M. hapla* species and other nematodes. (**A**,**B**) The specificity of *M. hapla* amplification products using LAMP. (**C**), The specificity of *M. hapla* amplification products using traditional PCR. Number 1–27 represents isolates of nematodes Mh1, Mh2, Mh3, Mh4, Mi1, Mi2, Mi3, Mi4, Mi5, Mi6, Me1, Me2, Me3, Me4, Mj1, Mj2, Mj3, Ma1, Ma2, Mg1, Mg2, Mm1, Mc1, MH, Pc, Dd, Hg, number 28 represents the negative control with no template DNA, M represents a DL2000 DNA size marker (ordinate values in bp).

Specificity of the LAMP was evaluated using 9 *Meloidogyne* species and 3 other plant nematodes (Table 1). The positive colour reactions were obtained with the DNA template from *M. hapla*, but were not observed in other nematode species (Fig. 3A). The LAMP amplifications were tested by LFD strips (Fig. 3B). The results indicated that the LAMP assay could distinguish *M. hapla* from closely related *Meloidogyne* species and other plant nematodes. The results were confirmed by an *M. hapla*-specific primer set, one band at 960 bp was detected in the four isolates of *M. hapla* (Fig. 3C).

### Sensitivity comparison of LAMP with conventional PCR

**Figure 4:**
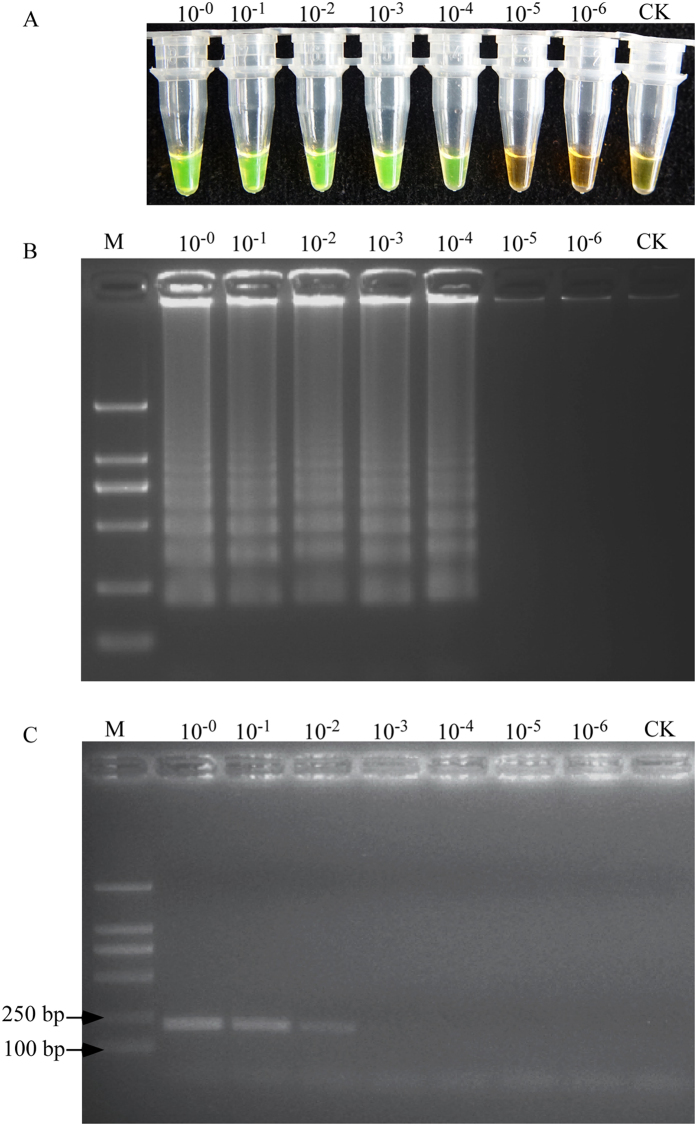
The sensitivity of the LAMP assay and the conventional PCR for detection of *M. hapla*. The two methods were carried out at the following, the negative control used water. The conventional PCR was performed with primers F3 and B3. (**A**) Sensitivity of the LAMP products detected by SYBR Green I fluorescence dye (**B**) Sensitivity of the LAMP products detected by gel electrophoresis. (**C**) Sensitivity of the conventional PCR products detected by gel electrophoresis. Concentrations of 10^−0^, 10^−1^, 10^−2^, 10^−3^, 10^−4^, 10^−5^ and 10^−6^ of single female nematode genomic DNA were used, CK represents no-template control. Lane M represents a DL2000 DNA size marker (ordinate values in bp).

A series of 10-fold dilutions of *M. hapla* DNA extracted from a single adult female were used to determine the sensitivity of the LAMP assay. Positive results were observed when at least 1 μl of the lysate was in the reaction mixture (that is, 5 × 10^−5^ of an adult female in the reaction mixture) (Fig. 4A,B). In a comparative analysis of the LAMP and conventional PCR assays, the LAMP assay was 100-fold higher sensitivity than the conventional PCR, which had a detection limit of 5 × 10^−3^ of single adult female lysates (Fig. 4C). No amplification was observed in the no-template control.

### LAMP analysis combined with FTA Technology

**Figure 5:**
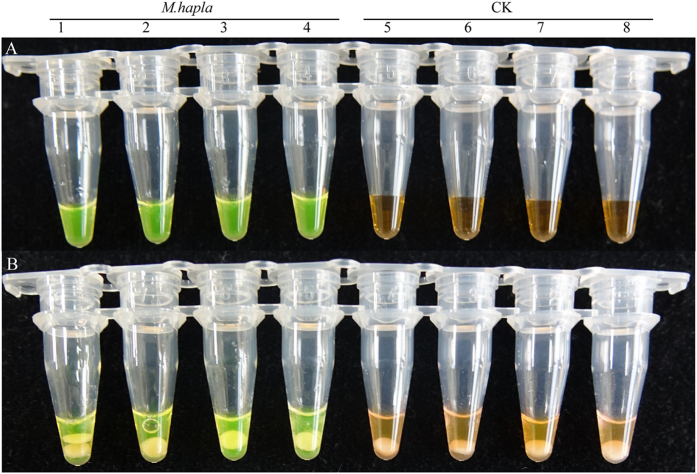
Comparison between the FTA technology and the standard DNA extraction procedure. (**A**) LAMP amplification with the standard DNA extraction procedure (**B**) LAMP amplification with the FTA technology. Lines 1–4 represent DNA isolated from *M. hapla* induced root gall, lines 5–8 represent DNA isolated from health root and used as negative control.

To prove the applicability of the FTA-LAMP assay for direct *M. hapla* detection from plant root galls, two different methods were compared using the DNA extracted from a single root gall. The FTA-based assay presented similar results to the conventional DNA isolation method (Fig. 5), indicating that the FTA technology has the potential to be used for detection of plant nematodes combine with the LAMP assay.

### Field evaluation of LAMP in infested plant root galls

**Figure 6:**
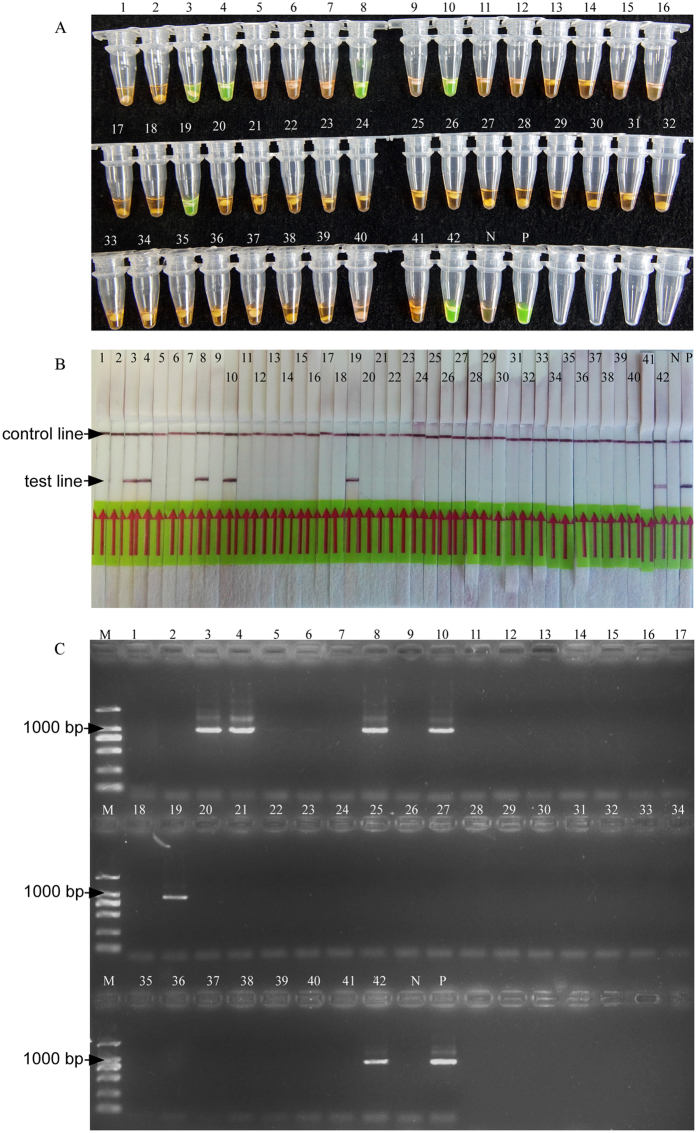
Application of LAMP and conventional PCR on field samples. The number of samples is identical to that in Supplementary Table 1. N; negative control. P; positive control. (**A**) LAMP products detected by SYBR Green I fluorescence dye (**B**) LAMP products detected by LFD strip. (**C**) Conventional PCR products detected by gel electrophoresis.

For the artificially inoculated plant root, 19 of the 20 (95%) replicated LAMP reactions occurred a positive result using *M. hapla* induced galls, whereas with the same crude DNA extracts, only 16 of the 20 (80%) samples were successfully amplified by PCR assay. The negative results were observed from other closely related *Meloidogyne* species induced galls and healthy roots (Table 2). Furthermore, positive results were presented in 6 of the 42 field samples using FTA-LAMP with detection rates of 100% (Fig. 6 and Supplemental Table 2). The results were confirmed by rDNA-ITS and morphological observation. In the negative samples, although there were other Meloidogyne species, no *M. hapla* specimens were detected.

## Discussion

*M. hapla* has a wide range of hosts, and can be distributed in various climate areas^29^. However, a rapid and precise diagnosis method is urgently required. Molecular techniques can help species identification, but all current techniques have limitations. Traditional *M. hapla* detection assays based on PCR methods^11^,^12^,^13^,^15^,^30^ are time-consuming and require sophisticated equipments as well as expertise. LAMP is a novel, simple, rapid and precise amplification method that can be adapted for diagnosing plant-parasitic nematodes, as previously reported^9^,^25^,^26^,^27^,^28^. In this study, we designed a set of five specific LAMP primers to detect *M. hapla*. The reaction can be completed in a water bath, and the amplified products can be detected visually by the naked eye rather than expensive instruments within 1 hour. Niu *et al*.^26^ developed a universal LAMP set (RKN-LAMP) that could be used to detect four common *Meloidogyne* species (*M. incognita, M. arenaria, M. javanica* and *M. hapla*), and *M. incognita*-specific LAMP set (Mi-LAMP), however, the *M. hapla*-specific LAMP assay was not recorded. In our study, the specific LAMP assay for detection of *M. hapla* was developed.

Several specific primers of *M. hapla* have been developed based on the ribosomal intergenic spacer (rDNA)^10^,^15^,^16^ and RAPD patterns^12^. Zijlstra *et al*.^14^ cloned and sequenced the ITS regions and found sufficient variability in those regions to separate *M. hapla* from *M. chitwoodi*. Then, the PCR primers were designed to separate *M. chitwoodi, M. fallax, M. hapla*, and *M. incognita* based on the ITS sequences^11^. In this study, we designed a set of five specific LAMP primers for *M. hapla* based on the ITS regions. LAMP based on these five primers is more specific compared to the conventional PCR pair-primers. Furthermore, the LFD strips were a great improvement to specificity and effectively avoided presenting false positives in the reactions. Additionally, the positive results were only present in four populations of *M. hapla* but did not appear in the other eight closely related *Meloidogyne* species, the test showed high specificity, but we tested a limited number of nematodes populations and did not assess other related species such as *M. chitwoodi* and *M. fallax*. Even the primers used in this study were selected in the ITS regions where there exist differences between *M. hapla* and other *Meloidogyne* species (Fig. 1) and had high specificity to avoid the risk of misdetection. More isolates of *M. hapla* and other related species should be examined using the LAMP method in the future.

Previous results found that the sensitivity of the LAMP assay was higher than conventional PCR^9^,^26^,^27^,^28^. In the present study, the detection limit was low to 1/200 000 nematode DNA, whereas the conventional PCR approach usually requires a single nematode, Williamson *et al*.^11^ designed the RAPD primers and Zijlstra *et al*.^12^ described the SCAR assay for the detection of *M. hapla*. The minimum amount of DNA that could be detected was a single juvenile nematode. In this study, pair-primers F3 and B3 were used for conventional PCR to detect *M. hapla*, and the sensitivity was 1/2000 nematode DNA (Fig. 4). Therefore, the detection limit of the LAMP was 100 times higher than that of PCR-based detection methods. The sensitivity of the LAMP was equal to *B. xylophilus*^25^, *M. enterolobii*^9^, *M. incognita*^26^, *R. similis*^27^ and *T. semipenetrans*^28^.

In a previous study, isolating nematodes from a Baermann funnel or direct picking nematodes from plant root galls required more time and specialized technique. The direct detection of plant nematodes using DNA from infected plant tissues has been published^27^,^31^,^32^,^33^,^34^, although those methods for preparing nucleic acids require toxic reagents and expansive instruments. In this study, direct extraction of DNA from plant root galls greatly improved the efficiency when using the FTA technology. This assay rarely reported in molecular plant nematology was not time-consuming and is using a nontoxic reagent. Marek *et al*.^35^ developed an FTA-based technology for the collection, long-term archiving and molecular analysis of three species of nematodes, including *Ditylenchus dipsaci, Heterodera schachtii* and *M. hapla* that involved PCR amplification. Our study demonstrated that the LAMP assay combined with FTA technology was fully applicable to plant nematode detection as well as the established conventional methods (Fig. 5). To the best of our knowledge, this is the first evidence of detecting plant nematodes using the LAMP assay combined with FTA technology, and the total detection time was shortened to one hour.

To evaluate the practical application of the FTA-LAMP for analyzing infected root galls, this method was tested on a collection from both artificially inoculated samples and field root galls. For artificially inoculated samples, 19 of the 20 (95%) *M. hapla*-induced root galls were successfully amplified. In contrast, only 16 of the 20 (80%) samples were successfully detected by PCR assay. Subsequently, the validity of the FTA-LAMP assay for *M. hapla* detection was also performed in 42 field samples (Fig. 6). All of the root galls infested with *M. hapla* were successfully detected by the FTA-LAMP assay with a detection rate of 100%, which indicated the high potential of this method. Combined with similar observations from previous reports^9^,^25^, these results showed that this assay has great stability and sensitivity, and can overcome interference from various types of PCR inhibitors, such as humic acid, proteins and non-target DNA. Furthermore, the FTA-captured nematode DNA could be stored at room temperature for many years^35^, which means that the field samples could be collected and that DNA purification could be accomplished using the FTA protocol; then, those samples could be tested with the LAMP assay, which it is simple, rapid and reliable.

In conclusion, the present study developed a FTA-LAMP assay for *M. halpa* detection. This novel FTA-LAMP assay could easily be used as a more sensitive, specific, and practical method for directly detecting *M. halpa* in infected plant tissues compared to previous methods. It will be potentially useful for monitoring and managing of *M. halpa* in the field.

## Materials and Methods

### Biological materials

Nine *Meloidogyne* species and three other plant nematode species used in this study are listed in Table 1. *M. mali* and *M. camelliae* were intercepted by China entry-exit Inspection and Quarantine in imported plant material, *M. graminicola* were collected from Myanmar and China. All *Meloidogyne* species except *M. graminicola* were purified from single egg-mass and reared on the susceptible tomato *cv*. Jiafen No. 9. The *M. graminicola* was cultured on the Rice *cv*. Nipponbare. Non-*Meloidogyne* genus including *Pratylenchus coffeae, D. destructor* and *H. glycines* were collected from China and used to verify the specificity test. All populations had been identified and previously diagnosed by morphological characteristics, rDNA-internal transcribed spacer (ITS) and species-specific primers^11^,^12^. Detailed protocols were described as below.

### DNA extraction

The genomic DNA of nematode was extracted by two different methods, and a portion of the samples was completed as described in Ou *et al*.^36^. Another group of samples and root galls was isolated by an FTA card (Whatman, GE Healthcare, USA) using a process that as previously described^35^, the root gall was placed on the FTA card and crushed with a pestle, a 2 mm diameter piece of the FTA card containing biological material was manually isolated and transferred into a tube, the disk was washed with FTA purification solution (Whatman, GE Healthcare, USA) and Tris-EDTA (TE) buffer (10 mM Tris and 0.5 mM EDTA [pH 8.0]) and air dried, the disk was directly used as a template for LAMP amplification.

### PCR amplification

Primers for rDNA-ITS amplification were rDNA1 and rDNA2^37^, a *M. hapla* specific SCAR primer^11^,^12^ were used to evaluate the specificity of the LAMP assays (Supplementary Table 1). The LAMP outer primers (Mh-F3/B3) were used to detect the sensitivity of traditional PCR. Amplification was performed in a 25 μl reaction volume with 0.4 μmol each of forward and reverse primers (Mh0F/Mh1R and Mh-F3/Mh-B3, Supplementary Table 1), 2.5 μL 10×PCR buffer (TaKaRa), 2 μL dNTPs, 1U ExTaq, and 1 μL of DNA template, double distilled water added for a final volume of 25 μL. The amplification was carried out under the following cycling conditions: 94 °C for 5 min, then 35 PCR cycles of 94 °C for 30 second, 55 °C for 30 second, 72 °C for 30 second and final incubation at 72 °C for 10 min.

### Design of LAMP Primers

Sequences of the rDNA-ITS were selected as the candidate targets for LAMP primer design. The *M. hapla* rDNA-ITS sequences amplified in this study (GenBank accession No. JX024147 and JX024148) and the sequence of other related species including *M. minor* (GU432775.1), *M. graminicola* (HM623442.1), *M. incognita* (JQ405212.1), *M. arenaria* (AF387092.1), *M. javanica* (AY438555.1), *M. enterolobii* (JF309153.1), *M. hispanica* (JX885741.1), *M. chitwoodi* (JN157868.1) and *M. fallax* (JN157869.1) were downloaded from Genbank at the NCBI website and used to compare the diversity of the rDNA-ITS sequence of *Meloidogyne* populations by MEGA5.0^38^. The specific primers of LAMP were designed using the Primer Explorer V4 software (http://primerexplorer.jp) according to the rDNA-ITS sequence difference regions. Five primers were constructed: two outer primers (F3 and B3), a forward inner primer (FIP), a backward inner primer (BIP) and a loop backward primer (LB). FIP comprised the F1c sequence complementary to the F1 and F2 sequence. BIP consisted of the B1c sequence complementary to the B1 and B2 sequence (Fig. 1 and Supplementary Table 1).

### Optimization of the LAMP reaction

The LAMP reaction was performed according to the protocol published previously^19^ with a minor modification. In a brief, the procedure used a 25 μL LAMP reaction mixture containing 1.4 μM each of the inner primers FIP and BIP, 0.2 μM each of the outer primers F3 and B3, 0.8 μM of the LF primer (forward loop primer), (0, 0.4, 0.8, 1.2, 1.6, 2.0, 2.4 mM) of a dNTPs mix, (2.0, 3.0, 4.0, 5.0, 6.0, 7.0, 8.0 mM) MgSO_4_, (0, 0.1, 0.2, 0.3, 0.4, 0.5, 0.6 M) betaine, 8U of Bst DNA polymerase (New England Biolabs GmbH, USA), 2.5 μL of 10×Thermopol reaction buffer (20 mM Tris-HCl (pH 8.8, 25 °C), 10 mM KCl, 10 mM (NH_4_)_2_SO_4_, 2 mM MgSO_4_, 0.1% Triton X-100), 1 μL of genomic DNA solution, and double distilled water was added to reach a total volume of 25 μL. The reaction mixture was incubated at 60–65 °C for 30–90 min and terminated by incubating at 80 °C for 5 min. In this study, the concentrations of Mg^2+^, dNTPs, betaine and the reaction times were optimized.

### Analysis of LAMP products

The LAMP amplification results were detected with three methods: adding the fluorescent dye SYBR green I (Invitrogen, 1:1000 TE buffer) to the reaction mixture and visually inspecting the results with the naked eye or under UV light; a Lateral-flow dipstick (LFD) assay that as visually observed with naked eyes, and 2% agarose gel electrophoresis.

### Lateral-flow dipstick (LFD) assay

In the LAMP-LFD assay, the 5′ biotin-labeled inner primer FIP was used. A DNA probe labelled by FITC at the 5′ end was designed from the sequence between the F3 and B3 regions, and the other primers were designed using the same procedure as previously described (Supplementary Table 1). After the LAMP reaction, 4 μl of the FITC-labeled probe (20 pmol/μL) were added into the LAMP reaction solution and incubated at 63 °C for 5 min to hybridize. Then, 8 μl hybridized product was transferred into 100 μl assay buffer in the reaction well. The LFD Strip (Milenia Biotec, Germany) was dipped into the mixer for approximately 5 min to detect the amplicon-probe hybrid; the positive result showed a test line and a control line, whereas the negative control only had a control line.

### Specificity and sensitivity comparison of LAMP to conventional PCR

To detect specificity of the LAMP assay, genomic DNA isolated from several *Meloidogyne* spp. and other plant nematode species was compared (Table 2). Meanwhile, a set of *M. hapla*-specific PCR primers Mh-0F and Mh-1F were used to verify the accuracy of the LAMP assay^12^. Specificity tests were repeated three times.

To determine the LAMP sensitivity, a series of 10-fold dilution of single female *M. hapla* genomic DNA (10^0^, 10^−1^, 10^−2^, 10^−3^, 10^−4^, 10^−5^ and 10^−6^) was prepared and used for both the LAMP and traditional PCR assays. SYBR Green I dye and electrophoresis were used to detect the LAMP products. Sensitivity tests were repeated three times.

### Detection of *M. hapla* in artificially inoculated plant root galls

The susceptible tomato *cv*. Jiafen No. 9 seeds were plated in the 15 cm diameter pots containing autoclaved soil and cultured in an environment-controlled chamber at 25 °C and a 16-h light/8-h dark cycle. Four weeks after seeding, the roots were inoculated with *M. hapla, M. incognita, M. enterolobii, M. javanica* and *M. arenaria*, individually, as described by Atamian *et al*.^39^. The rice *Oryza sative* L *cv*. Nipponbare was also planted in sand soil and inoculated with *M. graminicola* as described by Haegeman *et al*.^40^. Thirty days post inoculation (dpi), the single gall was collected and used to extract genomic DNA with FTA technology as described above. The healthy roots were used as negative control. Every sample was repeated twenty times.

### Field evaluation of FTA-LAMP

To determine the practical application of the FTA-LAMP process in the field, 42 field samples, including root galls and soil in different regions across China, were collected and tested (Supplementary Table 2). DNA extraction from a single root gall by an FTA card and the LAMP reaction were performed as described above. The results were confirmed by both an rDNA-ITS assay as described by Vrain *et al*.^37^ and the morphological identification^41^. As a comparison, the purified DNA from *M. hapla* and sterilized water were used as positive and negative controls, respectively. Three independent tests were performed for each sample.

## Additional Information

**How to cite this article**: Peng, H. *et al*. Rapid, simple and direct detection of *Meloidogyne hapla* from infected root galls using loop-mediated isothermal amplification combined with FTA technology. *Sci. Rep.* **7**, 44853; doi: 10.1038/srep44853 (2017).

**Publisher's note:** Springer Nature remains neutral with regard to jurisdictional claims in published maps and institutional affiliations.

## Supplementary Material

Supplementary Table 1

Supplementary Table 2

## Figures and Tables

**Table 1:** Species or population of RKN used to assess specificity of LAMP and PCR.

Number	Species	Isolate	Origin of population	Host	ITS	PCR	LAMP	
							Gel	LFD
1	*Meloidogyne hapla*	Mh1	Haidian Beijing	Chinese rose	+	+	+	+
2		Mh2	kunming Yunan	Peanut	+	+	+	+
3		Mh3	Yantai Shandong	peanut	+	+	+	+
4		Mh4	Zaozhuan Shandong	pomegranate	+	+	+	+
5	*M. incognita*	Mi1	Daxing Beijing	Tomato	+	−	−	−
6		Mi2	Langfang Hebei	Tomato	+	−	−	−
7		Mi3	Hefei Anhui	Cucumber	+	−	−	−
8		Mi4	Miyun Beijing	Tomato	+	−	−	−
9		Mi5	Hohhot Inner Mongolia	Cucumber	+	−	−	−
10		Mi6	Zhengzhou Henan	Tobacco	+	−	−	−
11	*M. enterolobii*	Me1	Haikou Hainan	Pacara earpod	+	−	−	−
12		Me2	Sanya Hainan	Euphorbia tirucalli Linn	+	−	−	−
13		Me3	Guangzhou Guangdong	Tomato	+	−	−	−
14		Me4	Yuanmei Yunnan	Tomato	+	−	−	−
15	*M. javanica*	Mj1	Kunming Yunnan	Tomato	+	−	−	−
16		Mj2	Haikou Hainan	Weed	+	−	−	−
17		Mj3	Sanya Hainan	Banana	+	−	−	−
18	*M. arenaria*	Ma1	shouguang shandong	Tobacco	+	−	−	−
19		Ma2	Yantai Shandong	Peanut	+	−	−	−
20	*M. graminicola*	Mg1	Haikou Hainan	Rice	+	−	−	−
21		Mg2	Myanmar	Rice	+	−	−	−
22	*M. mani*	Mm1	Japan^a^	Japanese maple	+	−	−	−
23	*M. camelliae*	Mc1	Japan^a^	Tea tree	+	−	−	−
24	*M. hispanica*	MH	DangzhouHainan	Morinda of ficinalis	+	−	−	−
25	*Pratylenchus coffeae*	Pc	Haikou Hainan	*Pandanus*	+	−	−	−
26	*Ditylenchus destructor*	Dd	Tongshan jiangsu	Sweet potato	+	−	−	−
27	*Heterodera glycines*	Hg	Haerbin Heilongjiang	Soybean	+	−	−	−

^a^Intercepted by CIQ (China entry-exit Inspection and Quarantine) in imported plant material.

**Table 2:** Detection of *Meloidogyne hapla* in root galls using LAMP or conventional PCR.

Samples	Host	LAMP		PCR assay	
		Positive/trials	Positive rate	Positive/trials	Positive rate
*M. hapla*-induced galls	tomato	19/20	95%	16/20	80%
*M. incognita*-induced galls	tomato	0/20	0	0/20	0
*M. arenaria*-induced galls	tomato	0/20	0	0/20	0
*M. enterolobii*-induced galls	tomato	0/20	0	0/20	0
*M. javanica*-induced galls	tomato	0/20	0	0/20	0
*M. graminicola*-induced galls	rice	0/20	0	0/20	0
Healthy root	*tomato*	0/10	0	0/10	0
Healthy root	*rice*	0/10	0	0/10	0
