# Supplementary material for: Rapid, simple and direct detection of Meloidogyne hapla from infected root galls using loop-mediated isothermal amplification combined with FTA technology
Source: Sci Rep. 2017 Apr 3;7:44853. doi: 10.1038/srep44853 (PMC5377304; doi:10.1038/srep44853)
Supplement: Supplementary Table 1 [file srep44853-s1.pdf]

Rapid, simple and direct detection of *Meloidogyne hapla* from infected root galls using loop-mediated isothermal amplification combined with FTA technology

Huan Peng[1], Haibo Long[2], Wenkun Huang[1], Jing Liu[1], Jiangkuan Cui[1], Lingan Kong[1], Xianqi Hu[3], Jianfeng Gu[4], Deliang Peng[1*]

Supplemental Table 1. Nucleotide sequences of the primers

Primer set	name	Sequences(5′-3′)	Usage	References
ITS	rDNA1	TTGATTACGTCCCTGCCCTTT	rDNA-ITS	[37]
	rDNA2	TTTCACTCGCCGTTACTAAGG	universal	
			primer	
LAMP	Mh-F3	GAATATGAGGTGACATGTTAGG	*M.hapla*	This study
	Mh-B3	TCAATGTTTCTGCAGTTCG	specific	
	Mh-FIP	TGAAAAAAATATTGCTGGCGTC-C	LAMP	
		ACCTTAATCGGGTTTAAGACT		
	Mh-BIP	TCTATCCTTATCGGTGGATCACT-		
		CCACAAATTATCGCAGTTAGCT		
	Mh-LB	GGCTCGTGGATCCATGAAGAACG		
	Mh-FITC-	CTCGTGGATCCATGAAGAAC		
	Probe			
MH0F/1R	MH0F	CAGGCCCTTCCAGCTAAAGA	*M.hapla*	[12]
	MH1R	CTTCGTTGGGGAACTGAAGA	specific	
			PCR	
